# Nrf2 is overexpressed in pancreatic cancer: implications for cell proliferation and therapy

**DOI:** 10.1186/1476-4598-10-37

**Published:** 2011-04-13

**Authors:** Adam Lister, Taoufik Nedjadi, Neil R Kitteringham, Fiona Campbell, Eithne Costello, Bryony Lloyd, Ian M Copple, Samantha Williams, Andrew Owen, John P Neoptolemos, Chris E Goldring, B Kevin Park

**Affiliations:** 1 MRC Centre for Drug Safety Science, Department of Molecular and Clinical Pharmacology, Institute of Translational Medicine, University of Liverpool, UK; 2 The Liverpool NIHR Pancreas Biomedical Research Unit. The Liverpool Experimental Cancer Medicine Centre, Liverpool CR-UK Centre and Division of Surgery and Oncology, University of Liverpool, UK; 3 The Liverpool CR-UK Centre, Division of Pathology, University of Liverpool, UK; 4 Applied Cancer Biology Group, Liverpool CR-UK Centre and Division of Surgery and Oncology, University of Liverpool, UK

## Abstract

**Background:**

Nrf2 is a key transcriptional regulator of a battery of genes that facilitate phase II/III drug metabolism and defence against oxidative stress. Nrf2 is largely regulated by Keap1, which directs Nrf2 for proteasomal degradation. The Nrf2/Keap1 system is dysregulated in lung, head and neck, and breast cancers and this affects cellular proliferation and response to therapy. Here, we have investigated the integrity of the Nrf2/Keap1 system in pancreatic cancer.

**Results:**

Keap1, Nrf2 and the Nrf2 target genes AKR1c1 and GCLC were detected in a panel of five pancreatic cancer cell lines. Mutation analysis of *NRF2 *exon 2 and *KEAP1 *exons 2-6 in these cell lines identified no mutations in *NRF2 *and only synonomous mutations in *KEAP1*. RNAi depletion of Nrf2 caused a decrease in the proliferation of Suit-2, MiaPaca-2 and FAMPAC cells and enhanced sensitivity to gemcitabine (Suit-2), 5-flurouracil (FAMPAC), cisplatin (Suit-2 and FAMPAC) and gamma radiation (Suit-2). The expression of Nrf2 and Keap1 was also analysed in pancreatic ductal adenocarcinomas (n = 66 and 57, respectively) and matching normal benign epithelium (n = 21 cases). Whilst no significant correlation was seen between the expression levels of Keap1 and Nrf2 in the tumors, interestingly, Nrf2 staining was significantly greater in the cytoplasm of tumors compared to benign ducts (P < 0.001).

**Conclusions:**

Expression of Nrf2 is up-regulated in pancreatic cancer cell lines and ductal adenocarcinomas. This may reflect a greater intrinsic capacity of these cells to respond to stress signals and resist chemotherapeutic interventions. Nrf2 also appears to support proliferation in certain pancreatic adenocarinomas. Therefore, strategies to pharmacologically manipulate the levels and/or activity of Nrf2 may have the potential to reduce pancreatic tumor growth, and increase sensitivity to therapeutics.

## Introduction

Pancreatic cancer is a leading cause of cancer-related deaths in the US and in Europe [1]. It carries a dismal prognosis, which is attributed in part to a high level of resistance to chemotherapeutic drugs [2]. For the vast majority of patients, the disease is at an advanced stage when diagnosed, and chemotherapy in the form of gemcitabine is the standard of care. Recent evidence suggests that combining gemcitabine with other agents, such as erlotinib or capecitabine, may provide greater benefit [3,4]. A small minority of patients (10-20%) can avail of potentially curative surgery, and for these patients the outlook is better [5,6]. Nonetheless, the overall survival rate of pancreatic cancer patients remains very poor. The mechanisms of drug uptake, DNA repair and apoptosis have all been proposed to contribute to the resistance of pancreatic cancer cells to chemotherapy [7]. Moreover, a recent study using a genetically-engineered mouse model of pancreatic cancer revealed that treatment failure could be attributed to inefficient gemcitabine delivery to tumor cells, likely due to poor vascularisation of the tumor [8]. A deeper understanding of the mechanisms of chemotherapy resistance in pancreatic cancer cells may allow the development of more targeted treatment options.

The Nuclear factor erythroid 2-related factor 2 (Nrf2)/Kelch-like ECH-associated protein 1 (Keap1) system represents an important mechanism by which mammalian cells can sense and adapt to chemical and oxidative stresses [9-11]. Normally, Keap1 targets Nrf2 for ubiquitylation, leading to its proteasomal degradation [12]. In response to chemical or oxidative stress, the interaction between Nrf2 and Keap1 is perturbed, resulting in the stabilization and nuclear accumulation of Nrf2 [11,13]. Nrf2 localised in the nucleus interacts with antioxidant response elements in the promoter regions of a plethora of genes coding for phase 2 detoxifying enzymes (e.g. glutathione-S-transferases and NAD(P)H quinone oxidoreductase), antioxidant proteins (e.g. glutathione synthetic enzymes) and transporters (e.g. ABCC2, ABCC3, ABCG2 and x_c_^- ^subunit) [14-18].

Elevated Nrf2 levels have been observed in head and neck [19], gall bladder [20] and lung cancer [21], and evidence indicates that a dysregulated Nrf2/Keap1 system may protect against the deleterious effects of oxidative stress, whilst also conferring properties of enhanced cellular proliferation and a drug-resistant phenotype, in certain cancers [20,22,23], effectively acting as a double-edged sword [22]. Here we have investigated the integrity of the Nrf2/Keap1 system in pancreatic cancer.

## Results

### Delineation of the Nrf2/Keap1 system in five pancreatic cancer cell lines

**Figure 1:**
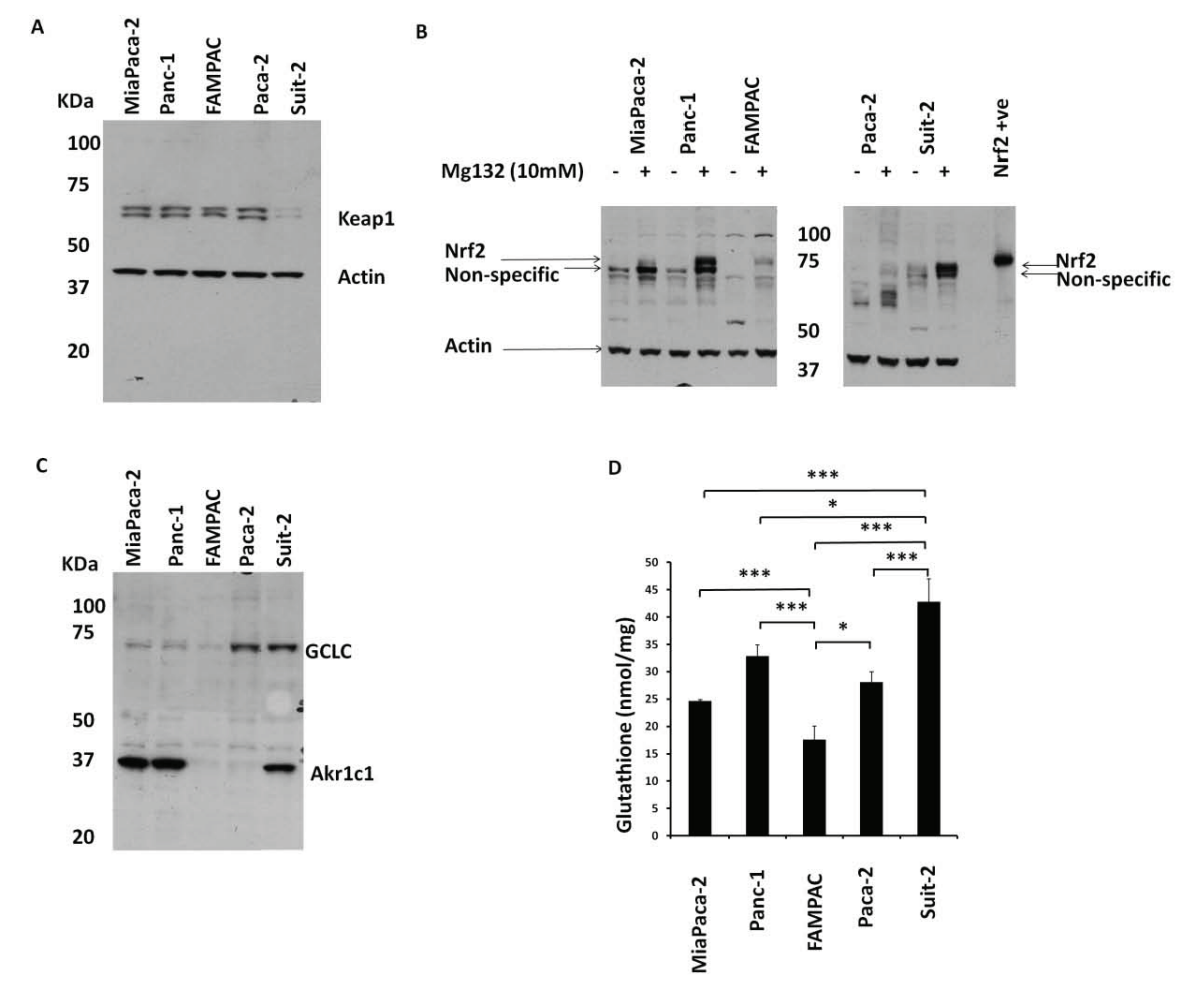
**Basal expression levels of Keap1, Nrf2 and Nrf2-regulated genes GCLC and AKR1c1 and GSH amongst a panel of pancreatic cancer cell lines**. A, Immunoblot detection of basal Keap1 protein in MiaPaca-2, Panc-1, FAMPAC, Paca-2 and Suit-2 cells. B, Immunoblot detection of basal Nrf2 in cells untreated or treated with the proteosome inhibitor MG132 (10 μM) for 2 h in order to visualize Nrf2 protein. The band labelled 'non-specific' was not depleted following transfection with 10 nM Nrf2-targeting siRNA Nrf2 (data not shown). Beta actin was used as a reference control for blots A and B. C, Immunoblot detection of basal GCLC and AKR1c1. D, Total basal glutathione levels. * = P < 0.05,** = P < 0.01,*** = P < 0.001.

In order to investigate the integrity of the Nrf2/Keap1 system in pancreatic cancer, we first examined the protein expression levels of Keap1 and Nrf2 across a panel of five human pancreatic cancer cell lines. In Miapaca-2, Panc-1, FAMPAC and Paca-2 cell lines, the basal expression levels of Keap1 were high, whilst the levels of Nrf2 were below the limit of detection. Conversely, the Suit-2 cell line had low levels of Keap1, and detectable levels of Nrf2, under basal conditions (Figure 1A and 1B). In all cell lines the proteasome inhibitor MG132 caused the stabilization of Nrf2 (Figure 1B), indicating that the classical mechanism of Nrf2 degradation exists in these cells.

To examine the correlation between the protein expression of Nrf2 or Keap1 and the abundance of their respective mRNA amongst the panel of cell lines, the copy numbers of Nrf2 and Keap1 mRNA were quantified by RTPCR (Additional file 1, Figure S1A). Significantly higher levels of Keap1 mRNA were detected in FAMPAC cells compared to MiaPaca-2 and Suit-2. These data indicate a correlation between Keap1 mRNA and protein levels in FAMPAC (high levels of Keap1) and Suit-2 (low levels of Keap1) cells, but reveal a lack of correlation in MiaPaca-2 cells (low Keap1 mRNA and high Keap1 protein) (Figure 1A and Additional file 1, Figure S1A). No significant differences were detected in the mRNA copy number of Nrf2 between the five cell lines (Additional file 1, Figure S1B), indicating that post-translational factors underpin the different levels of expression of Nrf2 protein across this panel of cells.

To relate the protein expression levels of Nrf2 with its basal activity, we examined the protein levels of two Nrf2 target genes, AKR1c1 and GCLC, as well as the product of GCLC activity, glutathione (GSH). FAMPAC cells expressed low basal AKR1c1 and GCLC (Figure 1C), correlating with their high Keap1 and low Nrf2 expression levels. Suit-2 cells expressed high levels of AKR1c1 and GCLC (Figure 1C), correlating with their low Keap1 and high Nrf2 expression levels. Additionally, total cellular GSH levels were significantly lower in FAMPAC cells compared to Suit-2 cells (Figure 1D). Interestingly, both MiaPaca-2 and Panc-1 cells expressed high levels of AKR1c1, and the Paca-2 cells expressed high levels of GCLC (Figure 1C), further indicating a lack of correlation between the expression levels of Keap1, Nrf2 and Nrf2 target genes, and as such a dysregulation of the Nrf2/Keap1 system, in these cell lines.

In order to explore the potential mechanisms underlying the dysregulation of the Nrf2/Keap1 system in the pancreatic cancer cell line panel, we sequenced the protein-coding exons 2-6 of the *KEAP1 *gene and exon 2 of *NRF2*, which have been shown to contain functionally relevant SNPs in other cancer types [24]. Heterozygotic *KEAP1 *gene mutations were observed in the MiaPaca-2 and Panc-1 cells (data not shown), although all were synonomous. *NRF2 *exon 2 was wild-type in all the cell lines (data not shown). Additionally, we analyzed the publicly-available transcript sequencing data from 24 pancreatic cancer samples, described by Jones *et al *[25]. Neither non-synonomous mutations nor copy number alterations were detected within the *NRF2 *or *KEAP1 *genes in these tumors. Taken together, these results indicate that post-translational factors contribute to the dysregulation of the Nrf2/Keap1 system in a subset of pancreatic cancer cell lines.

### Functional examination of the Nrf2/Keap1 system in pancreatic cancer cell lines

**Figure 2:**
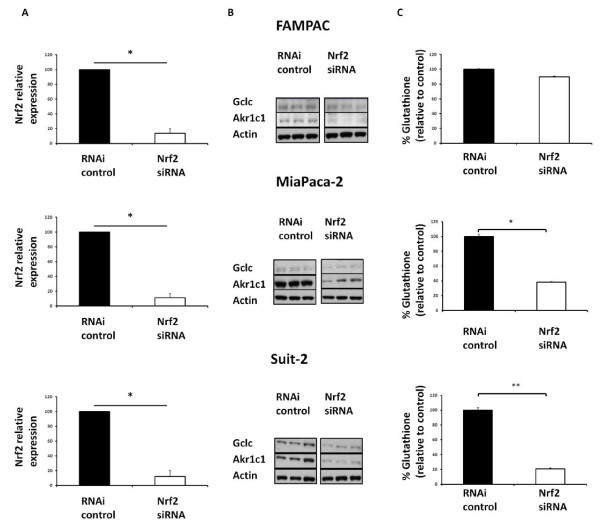
**Response of Nrf2-target genes to siRNA depletion of Nrf2 in FAMPAC, MiaPaca-2 and Suit-2 pancreatic cancer cells**. Cells were transfected with 10 nM Nrf2-targeting siRNA, or Stealth RNAi control, for 96 h. A, qRTPCR analysis of relative Nrf2 mRNA expression. GAPDH was used as a reference control. B, Immunoblot detection of GCLC and AKR1c1 in three parallel experiments. Beta-actin is used as reference control. C, Total glutathione levels. Data are the means ± S.D. of four discrete experiments. * = P < 0.05,** = P < 0.01.

We next explored the function of the Nrf2/Keap1 system in three pancreatic cancer cell lines showing extremes of Nrf2/Keap1 expression; Suit-2 (lowest Keap1 and highest Nrf2), FAMPAC (highest Keap1 and lowest Nrf2), and MiaPaca-2 (high Keap1 with a lack of correlation between levels of Nrf2 and expression of Nrf2 target genes) (Figure 1). siRNA depletion of Nrf2 caused a decrease in the expression levels of the Nrf2-regulated proteins GCLC and AKR1c1 in all three cell lines (Figure 2B and Additional file 2, Figure S2). Total GSH levels were significantly decreased, 96 h following Nrf2 siRNA transfection, in MiaPaca-2 and Suit-2 cell lines (Figure 2C). Significant downregulation of the Nrf2 target genes HO-1, MRP5 and BCRP was also observed in the Suit-2 cells following siRNA depletion of Nrf2 (Additional file 3, Figure S3). There were no significant changes in GSH following siRNA depletion of Nrf2 in the FAMPAC cell line (Figure 2C), which exhibits low basal Nrf2 expression and activity (Figure 1). Importantly, siRNA depletion of Keap1 resulted in an increase in the protein levels of Nrf2 and its target genes AKR1c1 and GCLC in both FAMPAC and Miapaca-2 cells (Additional file 4, Figure S4). These data suggest that, even in pancreatic cell lines in which the Nrf2/Keap1 system appears to be dysregulated, the pathway does retain its functional integrity.

### Nrf2 regulates pancreatic cancer cell proliferation

**Figure 3:**
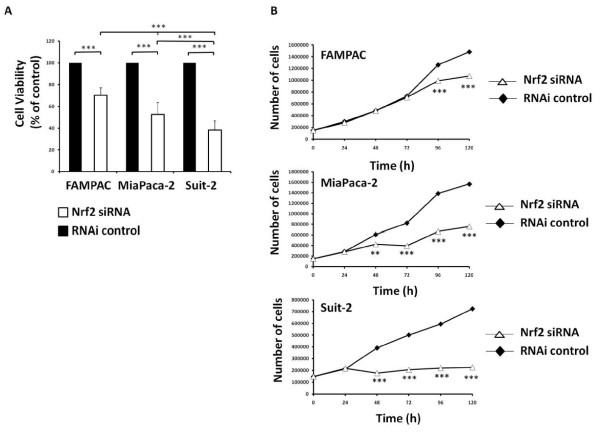
**Effect of siRNA depletion of Nrf2 on proliferation of FAMPAC, MiaPaca-2 and Suit-2 pancreatic cancer cells**. Cells were transfected with 10 nM Nrf2-targeting siRNA, or Stealth RNAi control, for up to 120 h. A, Cell survival was measured at 120 h using the MTS test. Data is shown as cell viability versus Stealth RNAi control. B, Cell numbers were quantified at the indicated time points using Trypan blue staining. Data are the means ± S.D. of four discrete experiments. ** = P < 0.01,*** = P < 0.001.

In light of recent reports that Nrf2 regulates cancer cell proliferation [22,23], and given that the Nrf2/Keap1 system is functional in Suit-2, Miapaca-2 and FAMPAC pancreatic cancer cells, we examined the role of Nrf2 in determining the rate of proliferation of these cells. siRNA depletion of Nrf2 in Suit-2 cells was accompanied by a > 60% decrease in viability compared to cells transfected with scrambled siRNA (Figure 3A). MiaPaca-2 and FAMPAC cells, which display intermediate and low levels of Nrf2 activity, respectively, exhibited intermediate or small decreases in viability following depletion of Nrf2 (Figure 3A). Trypan blue staining of cells over a 120 h timecourse revealed that siNrf2 depletion of Nrf2 caused Suit-2, MiaPaca-2 and FAMPAC cells to proliferate at a reduced rate, compared to cells transfected with non-targeting scrambled siRNA (Figure 3B). The effect was most pronounced in Suit-2 cells, which express the highest basal level of Nrf2, and least pronounced in FAMPAC cells, which express the lowest basal level of the transcription factor (Figure 3B). Importantly, siRNA depletion of Keap1 significantly increased the rate of proliferation of FAMPAC cells, which have high Keap1 protein levels and very low Nrf2 activity (Additional file 5, Figure S5). These results indicate that Nrf2, at least partly, contributes to the rate of proliferation of pancreatic cancer cells.

**Figure 4:**
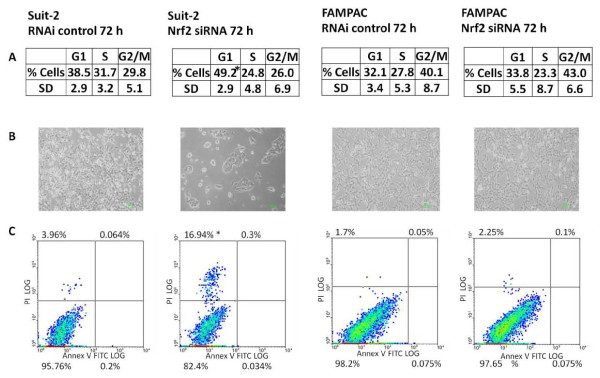
**Effect of siRNA depletion of Nrf2 on cell cycle progression and apoptosis status of Suit-2 and FAMPAC pancreatic cancer cells**. Cells were transfected with 10 nM Nrf2-targeting siRNA, or Stealth RNAi control, for 72 h. A, Flow cytometric analysis of cell cycle status in propidium iodide stained cells. Data are the means ± S.D of four discrete experiments. * = P < 0.05. B, Micrographs depicting cells analysed in A. C, Flow cytometric analysis of apoptotic status in Annexin 5/propidium iodide stained cells.

In order to further define the role of Nrf2 in determining the proliferation rate of pancreatic cancer cells, propidium iodide staining was employed to examine the cell cycle status of Suit-2 and FAMPAC (i.e. cells that exhibit high and low basal levels of Nrf2 activity, respectively) following siRNA depletion of Nrf2. In Suit-2 cells transfected with scrambled siRNA for 72 h, an approximately equal number of cells were found in each stage of the cell cycle (Figure 4A). In contrast, Suit-2 cells treated with Nrf2 siRNA for the same period of time showed a significant increase in the proportion of cells in G1 phase (Figure 4A). In contrast, the number of FAMPAC cells in the G1 phase did not differ following siRNA depletion of Nrf2 (Figure 4A). These results suggest that Nrf2 is important for progression through the G1 phase of the cell cycle in Suit-2 cells, which express high levels of the protein. However, in FAMPAC cells, in which Nrf2 is constitutively repressed, proliferation is not constrained by the requirement for Nrf2, and hence Nrf2 depletion has little effect on the rate of progression through the cell cycle (Figure 4A) or the overall rate of proliferation (Figure 3B).

In order to investigate whether Nrf2 expression altered the apoptotic status of the Suit-2 and FAMPAC cell lines, annexin-5/propidium iodide dual staining was performed 72 h following transfection with Nrf2-targeting siRNA. There was no evidence of apoptosis in the Suit-2 or FAMPAC cell lines (Figure 4C), which may reflect the late time point at which the assay was performed or that neither cell line expresses functional p53 protein [26,27]. Taken together, these results suggest that Nrf2 regulates the rate of proliferation and cell cycle progression in pancreatic cancer cells exhibiting high basal levels and activity of Nrf2, such as Suit-2.

### Nrf2 enhances chemo- and radioresistance in pancreatic cancer cells

**Figure 5:**
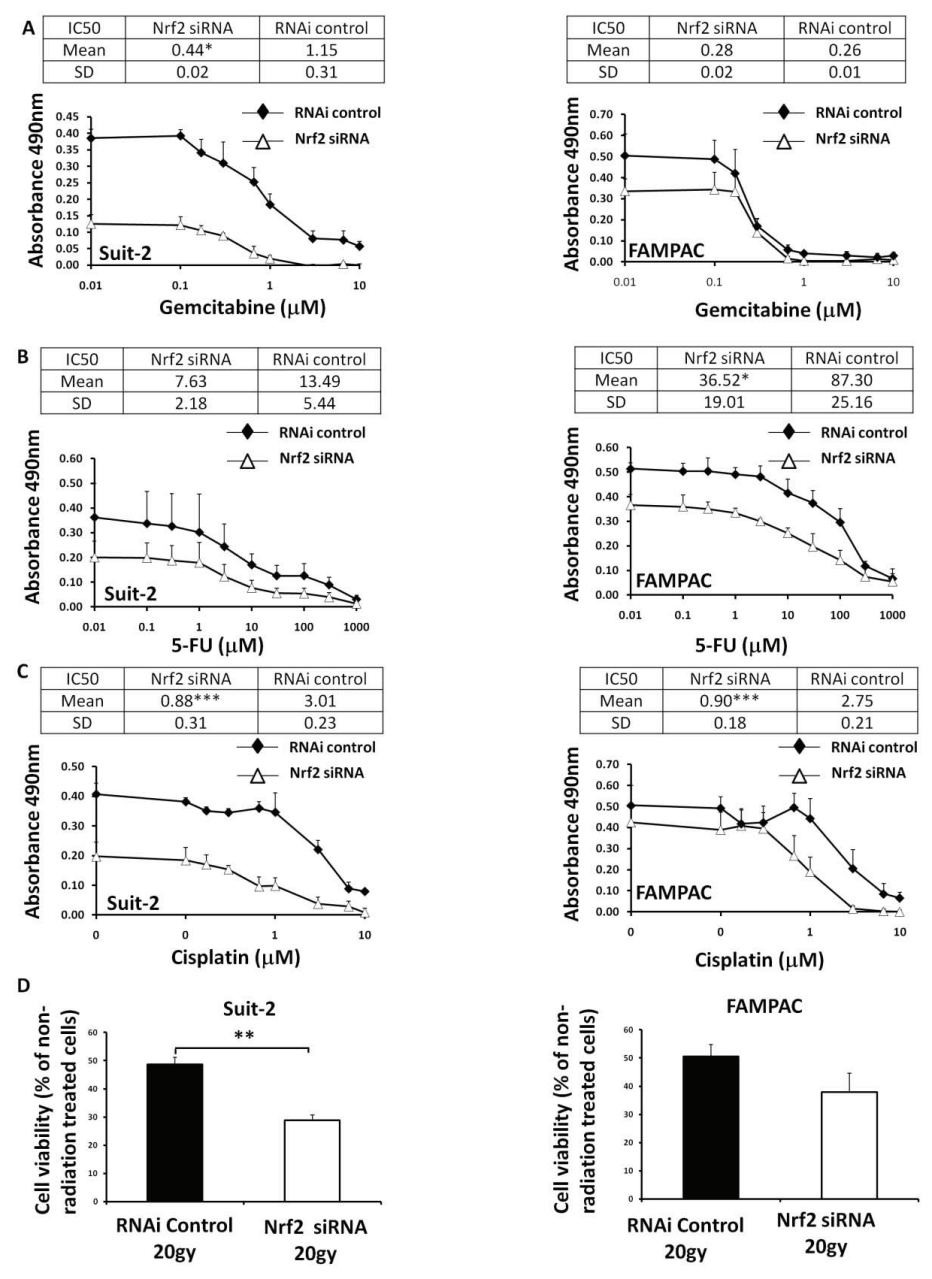
**Effect of siRNA depletion of Nrf2 on sensitivity of Suit-2 and FAMPAC pancreatic cancer cells to chemo- and radiotherapies**. Cells were transfected with 10 nM Nrf2-targeting siRNA, or Stealth RNAi control, for 48 h followed by exposure to gemcitabine (A), 5 flurouracil (5-FU) (B), cisplatin (C) or gamma radiation (D) for 72 h, at the indicated concentrations. Cell viability was measured using the MTS assay. Graphs represent data as a surviving fraction versus non-drug treated Stealth RNAi control transfected cells. IC50 values are expressed as μM. Data are the means ± S.D. of four discrete experiments. * = P < 0.05,** = P < 0.01,*** = P < 0.001.

Nrf2 has been shown to, at least partly, determine the sensitivity of cancer cells to chemotherapeutic agents [20-23]. We therefore examined of the role of Nrf2 in determining the sensitivity of Suit-2 and FAMPAC cells to the chemotherapeutic agents gemcitabine, 5-FU and cisplatin, as well as to gamma irradiation. siRNA depletion of Nrf2 elicited a significant increase in sensitivity to gemcitabine (2.6 fold; Figure 5A) and cisplatin (3.4 fold; Figure 5C), but had no discernable effect on sensitivity to 5-FU (Figure 5B), in Suit-2 cells. In FAMPAC cells, siRNA depletion of Nrf2 had no effect on sensitivity to gemcitabine (Figure 5A), but did cause a significant increase in sensitivity to 5-FU (2.3 fold; Figure 5B) and cisplatin (3.1 fold; Figure 5C). Depletion of Nrf2 caused a decrease in the viability of Suit-2 and FAMPAC cells following exposure to gamma radiation (Figure 5D), although the effect on FAMPAC cells was not statistically significant. Taken together, these results indicate that Nrf2 plays a role in determining the sensitivity of pancreatic cancer cells to chemotherapeutic agents.

### Expression of Nrf2 is increased in pancreatic cancer tumors

**Figure 6:**
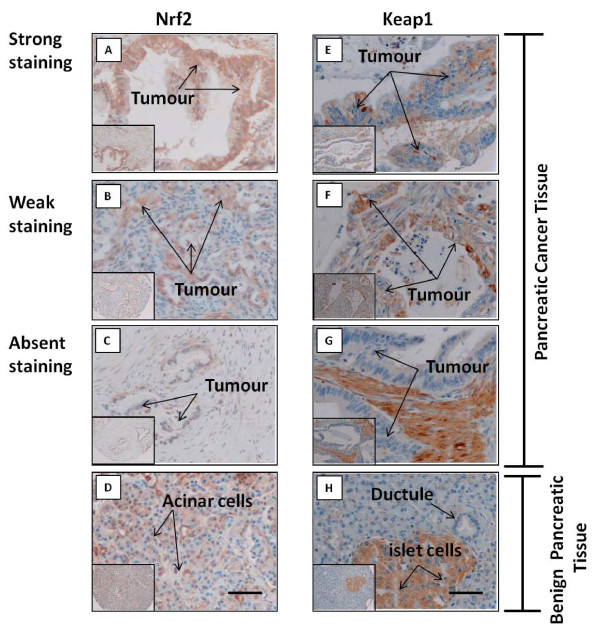
**Immunohistochemical analysis of Nrf2 and Keap1 in pancreatic tissues**. A and B, Pancreatic cancer tissue showing strong and weak Nrf2 levels, respectively. E and F, Pancreatic cancer tissue showing Keap1 expression. C and G, Pancreatic cancer tissue showing absence of detectable Nrf2 and Keap1, respectively. D and H, Benign pancreatic tissue showing Nrf2 expression in ductal cells and acinar cells (D) and absence of Keap1 in ductal cells, with positive islet cells (H). Scale bars = 50 μm.

Following our delineation of the Nrf2/Keap1 system in pancreatic cancer cell lines, we examined the expression levels of Nrf2 (Figure 6A-D) and Keap1 (Figure 6E-H) in human pancreatic tumor tissues, using immunohistochemistry. Cytoplasmic Nrf2 was detected in 93% (n = 53/57) of tumors, with strong staining (score > 45, the optimum cut-off value for discriminating between strong and weak staining was determined by the receiver operating characteristic curve (ROC curve)) observed in 84% of cases (n = 48/57). By contrast, whilst cytoplasmic Nrf2 staining was observed in a high percentage of matching benign ducts (86%, n = 18/21), staining was predominantly weak (66%; 14/21) with strong staining observed in 19% (4/21) of cases only. The increased cytoplasmic Nrf2 expression in tumors was statistically significant (P < 0.0001, Mann-Whitney U-test). Although 53% (n = 30/57) of patients had detectable Nrf2 in the nuclear compartment of tumor cells, no difference in nuclear Nrf2 levels was observed between tumor and benign epithelium (P = 0.44, Mann-Whitney U-test), In order to appraise the overall Nrf2 levels (cytoplasmic and nuclear) in tumors, compared to their matched benign tissue, Nrf2 stained tissues were categorised into four distinct groups: i) high cytoplasmic Nrf2 and high nuclear Nrf2 (H^C^/H^N^), ii) high cytoplasmic Nrf2 and low nuclear Nrf2 (H^C^/L^N^), iii) low cytoplasmic Nrf2 and high nuclear Nrf2 (L^C^/H^N^) and iv) low cytoplasmic Nrf2 and low nuclear Nrf2 (L^C^/L^N^). Notably, the majority of the tumors (47.6%; 10/21) expressed high cytoplasmic Nrf2 and high nuclear Nrf2 (H^C^/H^N^), whereas the majority of the benign ducts (47.6%; 10/21) expressed low cytoplasmic Nrf2 and low nuclear Nrf2 (L^C^/L^N^) (Additional file 6, Figure S6).

Keap1 was detected in 30% of tumors only (n = 19/63), and found exclusively in the cytoplasm (Figure 6E-H). Keap1 was not detected in benign ductal epithelium (n = 0/21). There was no correlation between the presence of Keap1 and the levels of either cytosolic or nuclear Nrf2 in the pancreatic tumors (P = 0.47 and P = 0.86, respectively, Mann-Whitney U-test), indicating that the dysregulated Nrf2/Keap1 phenotype observed in some pancreatic cancer cell lines is also apparent in primary tumors. We found no significant association between the levels of Nrf2 (cytoplasmic or nuclear) or Keap1 in the pancreatic tumors and various clinicopathological parameters, (Additional file 7 &8, Tables S1 & S2).

## Discussion

Nrf2 controls a battery of genes that protect cells from chemical and oxidative stresses, and a number of Nrf2-regulated genes have been reported to be overexpressed in pancreatic cancer cells [28-31]. Here, we have demonstrated a lack of consistent correlation between the basal expression levels of Keap1 and Nrf2 mRNA and protein, together with the activity of Nrf2, between pancreatic cancer cell lines, indicating that the Nrf2/Keap1 system may be dysregulated in pancreatic cancer.

In contrast to other cancers, we found no evidence for the existence of non-synonmous mutations in *NRF2 *or *KEAP1 *in our panel of cell lines, nor in publicly-available SAGE gene expression data from a panel of 24 advanced pancreatic adenocarcinomas [25]. However, the latter analysis revealed that in > 75% of the cancers, Nrf2 expression is at least 10-fold higher than in normal pancreatic ductal cells. A similar increase in Nrf2 protein expression in malignant, compared to benign, epithelium has been recently reported in a smaller cohort of pancreatic adenocarcinoma patients and non-matching controls [32]. Amongst our human pancreatic ductal tumor samples, the majority expressed high cytoplasmic Nrf2 and high nuclear Nrf2, whereas the majority of benign ducts expressed low cytoplasmic Nrf2 and low nuclear Nrf2. The increased cytoplasmic Nrf2 levels may reflect a greater intrinsic capacity of the tumor cells to respond to stress signals and resist chemotherapeutic agents. It is possible that the high expression of Nrf2 in the pancreatic adenocarcinoma tissues is due to the elevated expression of proteins that can increase the stability of Nrf2, such as Sequestosome-1 [33,34] and Prothymosin-α [35], by competing with Nrf2 for the Keap1 binding site. Other possible mechanisms include: Keap1 down-regulation via promoter methylation, which has been described in lung cancer [36], transcriptional up-regulation of the *NRF2 *gene (which although not identified here in the pancreatic cancer cell lines, may nevertheless play a role in the tumors), dysregulation of Nrf2 ubiquitylation and proteasomal degradation, and stabilization of Nrf2 as a result of chronic oxidative stress. The contribution of these and other factors to the relatively high expression levels of Nrf2 in pancreatic cancer cells should be further examined in order to better understand the contribution of Nrf2 levels to cellular phenotype.

Although we have demonstrated that cytoplasmic levels of Nrf2 are significantly elevated in pancreatic tumors compared to matching benign ducts, nuclear levels of Nrf2 do not appear to differ between the two tissue types. It is well established that oxidative stress represents a primary signal that causes cytoplasmic Nrf2 to accumulate within the nucleus [11]. Although cancer cells generally have high levels of ROS due to uncontrolled cellular proliferation [37], these oxidative stress signals may only be transiently present. In addition, the primary tumor samples used in this study were obtained by surgical resection prior to chemotherapy/radiation treatment, further restricting their exposure to oxidative stress. It is possible that the elevated cytoplasmic Nrf2 population observed in pancreatic cancer cells represents an increased capacity to sense and respond to perturbations in the cellular redox environment. It is likely that the nuclear localisation of this pool of Nrf2 requires further increases in ROS levels and/or the contribution of other factors. For example, the relative activities of nuclear localisation (NLS) and export signals (NES) within Nrf2, and the interaction between the transcription factor and the importin family of proteins, are believed to be important determinants of the subcelluar dynamics of Nrf2 [38,39]. Other factors, including the direct phosphorylation of Nrf2 [24], may be important. As such, further work is required to delineate the functional importance of the elevated cytoplasmic levels of Nrf2 in pancreatic cancer cells.

This study has provided evidence that Nrf2 can regulate the rate of proliferation and degree of resistance to chemotherapeutic agents in pancreatic cancer cells. Notably, suppression of the Nrf2 target HO-1 using siRNA has recently been shown to cause a decrease in proliferation, and an increase in sensitivity to gemcitabine, in pancreatic cancer tissue *in vitro *and *in vivo *[40]. Additionally, the efflux transporters BCRP and MRP5, which were shown here to be regulated by Nrf2 in pancreatic cancer cells, have been implicated in resistance to gemcitabine [32,41,42]. It has been reported that Nrf2 can be activated by 5FU, possibly as a result of drug-induced ROS production, in the Keap1-expressing human colon cancer HT-29 cell line [43]. Induction of Nrf2-regulated cell defence genes is associated with an increased resistance to 5FU, reversible by Nrf2-targeting siRNA [43]. We have demonstrated an increased sensitivity to 5FU following Nrf2 depletion in FAMPAC cells, although we did not observe a similar effect in Suit-2 cells that exhibit low levels of Keap1 and high levels of Nrf2. Interestingly, however, a recent study has revealed that siRNA depletion of Nrf2 in TGBC24TKB gall bladder cancer cells, in which Keap1 is not expressed and Nrf2 is constitutively present at high levels, can increase sensitivity to 5FU [20]. Clearly, the contribution of Nrf2 to chemotherapeutic drug resistance may be complicated, and indeed cell type specific, and further investigations are required to understand these mechanisms.

Exposure to gamma radiation causes ionization of water molecules in the cell, and increases the intracellular production of free radicals. Since Nrf2 is a key regulator of antioxidant defence, it is possible that the increased sensitivity to gamma radiation of Suit-2 cells following Nrf2 depletion is a result of the inability to mount a defence response in a perturbed redox environment. Indeed, the activity of Nrf2 has recently been shown to be important for the ability of prostate cancer cells to resist the cytotoxicity caused by exposure to radiation [44]. It may be valuable to further explore the role of Nrf2 in protecting against radiation-induced cell damage in pancreatic cancer. Our data, demonstrating that Nrf2 plays a role in the resistance of pancreatic cancer cells towards chemotherapeutic interventions, were generated following continuous exposure of cells *in vitro*. Future experiments will address the issue of short- and long-term exposure to these molecules, in order to begin to relate this data to clinically-relevant therapeutic strategies.

## Conclusions

The Nrf2/Keap1 system appears to be dysregulated, yet functional, in certain pancreatic cancer cell lines, and in primary pancreatic ductal adenocarcinomas. Furthermore, Nrf2 supports cellular proliferation and chemotherapeutic drug resistance in some of these cells. Pharmacological manipulation of the Nrf2/Keap1 signalling pathway has the potential to reduce the rate of growth of primary pancreatic tumors, and render them more susceptible to attack by chemotherapeutic agents.

## Materials and methods

### Reagents

DMEM was purchased from Lonza (Wokingham, UK). RPMI and foetal bovine serum was purchased from Gibco (Paisley, UK). Cis-diamminedichloroplatinum (cisplatin), streptomycin, penicillin, propidium iodide, RNase, RPMI-1640, RIPA buffer, all primers for sequencing and qRTPCR, Sybr green reagent, anti-rabbit horseradish peroxidise-conjugated secondary antibody and rabbit anti-actin primary antibody were purchased from Sigma (Poole, UK). CellTiter96 aqueous non-radioactive cell proliferation assay (MTS) and the ImProm-II Reverse transcription system were purchased from Promega (Southampton, UK). HotStarTaq reagents and QIAquick gel extraction kit were from Qiagen (Crawley, UK). Anti-goat horseradish peroxidise-conjugated secondary antibody was purchased from Dako (Ely, UK). Anti-sheep horseradish peroxidise-conjugated secondary antibody was purchased from Calbiochem (Nottingham, UK). Enhanced chemiluminescence was purchased from PerkinElmer (Beaconsfield, UK). Goat anti-Keap1 and rabbit anti-Nrf2 primary antibodies were purchased from Santa Cruz (Heidelberg, Germany). siRNA targeted against Nrf2 was purchased from Dharmacon (Lafayette, USA). Scrambled med GC RNA negative control, siRNA targeted against Keap1, TRIzol, Lipofectamine RNAiMAX and 4-12% Novex bis-tris polyacrylamide gels were purchased from Invitrogen (Paisley, UK). Rabbit anti-AKR1c1 primary antibody was a kind gift from Prof. John Hayes (University of Dundee, UK). Sheep anti-GCLC primary antibody was a kind gift from Dr. Lesley McLellan (University of Dundee, UK).

### Cell culture

MiaPaca-2, Panc-1, FAMPAC, Paca-2 and Suit-2 cell lines were maintained at 37°C in a 5% CO_2 _atmosphere in DMEM (MiaPaca-2, Panc-1 and Suit-2) or RPMI-1640 (FAMPAC and Paca-2), both supplemented with 10% fetal bovine serum, 100 U/mL penicillin and 100 ug/mL streptomycin.

### Immunoblotting

Whole cell lysates were obtained by lysing cells in RIPA buffer. Cell lysates were resolved on pre-cast 4-12% Novex bis-tris polyacrylamide gels. Separated proteins were transferred to nitrocellulose membranes, which were blocked for 30 min using 10% non-fat milk in tris-buffered saline. Membranes were probed for 24 h with anti-Nrf2, or for 1 h with anti-Keap1, anti-GCLC, anti-AKR1c1 or anti-actin primary antibodies. Membranes were washed and then probed for 1 h with the appropriate HRP-linked secondary antibody. Proteins were visualised by enhanced chemiluminescence using Hyperfilm ECL.

### Quantification of mRNA

Total RNA was extracted using TRIzol following the manufacturer's protocol. RNA quality and quantity was measured using a ND-1000 spectrophotometer (Nanodrop, Wilmington, USA). cDNA was synthesised using the ImProm-II Reverse transcription system. cDNA (50 ng) was analysed using qPCR using primers designed for Nrf2, HO-1, MRP5, BCRP and GAPDH (Additional file 9, Table S3) and Sybr green following the manufacturer's protocol. For siRNA-treated cells, GAPDH was used for normalisation. To measure the copy number of *NRF2/KEAP1 *mRNA per 50 ng of cDNA, standard curves (0-1,000,000 copies) were constructed from hNrf2 and hKeap1 plasmid expression vectors.

### Glutathione assay

Cell lines were grown for 24 h and total cellular glutathione (GSH) content was quantified as previously described [45].

### Sequencing of *KEAP1 *and *NRF2*

Genomic DNA from all five pancreatic cancer cell lines was amplified by PCR using HotStarTaq reagents and exon-specific primers for *KEAP1 *and *NRF2 *(Additional file 9, Table S3). Products were gel-purified using a QIAquick gel extraction kit and sequenced (Geneservice, Cambridge, UK). Sequences were subjected to a BLAST search against the wild type *KEAP1 *[NM_012289] and *NRF2 *[NM_006164] genomic sequences obtained from the NCBI database, and verified by manual analysis. Data relevant to the amplification, deletion and mutation status of *KEAP1 *and *NRF2 *was retrieved from the Jones *et al*. pancreatic cancer series [26].

### Genotyping of pancreatic cancer cell lines

The identities of all the cell lines used in this study were validated using the following approach: Genotyping was performed using PowerPlex-16 HS System (Promega) according to the manufacturer's instruction manual. Briefly, genomic DNA was isolated from pancreatic cancer cell lines using the Qiagen DNA mini kit. One ng of DNA was subjected to PCR reaction using PowerPlex-16 HS System (Promega), in accordance with the manufacturer's instructions. Detection of amplified fragments was carried out using Genetic Analyser (3130-Applied Biosystem) and GeneMapper software (Version 4.0).

### siRNA transfection

MiaPaca-2, FAMPAC and Suit-2 cells were seeded onto 96- and 6-well plates at 5 × 10^4 ^and 1 × 10^5 ^cells per well, respectively, and transfected using lipofectamine RNAiMAX with 10 nM of siRNA targeted against *NRF2, KEAP1 *or a scrambled RNA negative control. Cells were transfected for the specified time periods, depending on the subsequent analysis.

### Cell viability and proliferation assays

After 120 h transfection with siRNA molecules, cell viability was measured using the MTS assay. Alternatively, following siRNA transfection, cells were harvested at 24, 48, 72, 96, and 120 h, and viable cells were counted using Trypan blue staining.

### Analysis of cell cycle and apoptosis

FAMPAC and Suit-2 cells transfected with siRNA for 72 h were harvested, fixed in 70% ethanol solution and stored at 4°C overnight. Cells were then washed and treated with RNase (10 mg/ml) for 5 min, followed by the addition of propidium iodide (1 mg/ml). Cells were incubated in the dark for 30 min and the cell cycle was analysed using a Coulter Beckman flow cytometer. Data were processed using winMDI software. Apoptosis was measured using the Annexin V:FITC assay kit (AbD Serotec) according to the manufacturer's instruction manual. Briefly, FAMPAC and Suit-2 were transfected with siRNA for 72 h, stained with Annexin-5 for 10 min, then washed and stained with propidium iodide as described above. Apoptosis was detected using a Coulter Beckman flow cytometer.

### Chemo- and radiotherapy treatment

Forty-eight hours following siRNA transfection, cells were treated with the indicated concentrations of gemcitabine, 5-flurouracil or cisplatin, or 20 gy of gamma radiation (Gammacell 1000), for 72 h and cell viability was measured by MTS assay. Cell viability was expressed relative to the vehicle control-treated cells.

### Immunohistochemistry

Immunohistochemical staining was performed on a pancreatic ductal adenocarcinoma tissue microarray (TMA) containing matched duplicate non-malignant and malignant cores from 63 patients, who had undergone surgical resection at the Royal Liverpool University Hospital, UK, between 1994 and 2003. For 21 cancer cases, matching non-malignant cores contained benign ducts that could be evaluated. Immunohistochemical staining was performed as described previously [46], using primary antibodies directed against Keap1 (1:50 dilution) or Nrf2 (1:200 dilution). Isotype controls for both Nrf2 and Keap1 staining were also preformed (Additional file 10, Figure S7).

### Scoring and statistical analysis of immunohistochemically-stained tissue arrays

Scoring of the IHC slides was performed by two independent reviewers, one of whom (author Campbell) is a specialist gastrointestinal histopathologist. The information recorded for Nrf2 included the subcellular location and the intensity of staining (graded 0 = negative; 1 = weak; 2 = moderate; and 3 = strong) and the extent of staining (percentage of cells showing positive immunoreactivity: 0 - 100% of cells). For Nrf2, a score was assigned for each cellular compartment = the intensity of staining X the percentage of cells stained. Negative cases were defined as having a score of 0, weak cases, a score between 0 and 50 and strong cases had a score greater than 50. Keap1 staining was patchy and granular throughout the tumor. Tumors were therefore scored as either positive or negative for Keap1. Nrf2 or Keap1 immunohistochemical scores of benign and malignant cells were compared using the Mann-Whitney U-test. All statistical analyses were performed using Statview version 5.01. Further details on associations between Nrf2/Keap1 and clinicopathologic parameters are included in Additional file 7 &8, Tables S1 and S2.

### Statistical analysis

Data are expressed as the mean ± standard deviation of at least three independent experiments. The significance of differences within the data was assessed by one-way analysis of variance (ANOVA), with Tukey post-test for multiple comparisons. Student's paired t-test (parametric) or a Mann-Whitney test (non-parametric) was used for appropriate data sets. *P *values of < 0.05 were considered statistically significant.

## Abbreviations

Nrf2: Nuclear factor erythroid 2-related factor 2; Keap1: Kelch-like ECH-associated protein 1; AKR1c1: Aldo-keto reductase family 1 member C1; GCLC: Glutamate--cysteine ligase catalytic subunit; GAPDH: Glyceraldehyde 3-phosphate dehydrogenase; GSH: Glutathione; 5-FU: 5-flurouracil; SAGE: Serial *analysis *of gene expression; ROS: reactive oxygen species; HO-1: heme oxygenase 1.

## Conflict of interest

The authors declare that they have no competing interests.

## Authors' contributions

AL designed, performed and analyzed all cell line experiments and prepared the manuscript. CEG designed experiments, performed cell line sequencing and prepared the manuscript. TN performed immunohistochemistry of tumors. NK designed experiments and prepared the manuscript. FC analyzed and scored tumors. EC designed experiments and prepared the manuscript. IMC prepared the manuscript. SW performed additional experiments using RTPCR. BL analyzed SAGE data. AO prepared the manuscript. JPN resected tumor tissues and prepared the manuscript. BKP contributed to the conception and design of the study and the final editing of the manuscript. All the authors read and approved the final manuscript.

## Supplementary Material

Additional file 1 **Figure S1 - Quantification of Nrf2 and Keap1 mRNA abundance in pancreatic cancer cell lines**. cDNA was synthesised from isolated RNA from five pancreatic cancer cell lines. Nrf2 and Keap1 mRNA levels were quantified by RTPCR using plasmids containing hNrf2 and hKeap1 cDNA as standards. Click here for file

Additional file 2 **Figure S2 - Immunoblots depicting the area between 20-100 KDa to demonstrate specificity of antibodies used in the study**. Cells were transfected with 10 nM Nrf2-targeting siRNA, or Stealth RNAi control, for 96 h in three parallel experiments. A, Immunoblot detection of Keap1. Beta-actin is used as reference control. B, Immunoblot detection of GCLC and AKR1c1. Click here for file

Additional file 3 **Figure S3 - Quantification of HO-1, BCRP and ABCC5 mRNA levels in Suit-2 cells following siRNA depletion of Nrf2**. Cells were transfected with 10 nM Nrf2-targeting siRNA, or Stealth RNAi control, for 96 h. mRNA levels were quantified by qRTPCR. GAPDH was used as a reference control. Data are the means ± S.D. of four discrete experiments. * = P < 0.05, ** = P < 0.01. Click here for file

Additional file 4 **Figure S4 - siRNA depletion of Keap1 in FAMPAC and Suit-2 cells**. Cells were transfected with 10 nM Keap1-targeting siRNA, or Stealth RNAi control, for 96 h. Immunoblot detection of Keap1, Nrf2, GCLC and AKR1c1. Beta-actin is used as reference control. Click here for file

Additional file 5 **Figure S5 - Effect of siRNA depletion of Keap1 on viability of FAMPAC cells**. Cells were transfected with 10 nM Keap1-targeting siRNA, or Stealth RNAi control, for 120 h. Cell survival was measured using the MTS test. Data is shown as cell viability versus Stealth RNAi transfected control. *** = P < 0.001. Click here for file

Additional file 6 **Figure S6 - Distribution of Nrf2 staining in the cytoplasm and nucleus of tumors and matching benign cores**. Histograms showing Nrf2 stained tissues (n = 21 tumor cases and matched benign tissue) categorised into 4 distinct groups, i.e. those containing: i) high cytoplasmic Nrf2 and high nuclear Nrf2 (H^C^/H^N^), ii) high cytoplasmic Nrf2 and low nuclear Nrf2 (H^C^/L^N^), iii) low cytoplasmic Nrf2 and high nuclear Nrf2 (L^C^/H^N^) and iv) low cytoplasmic Nrf2 and low nuclear Nrf2 (L^C^/L^N^). Click here for file

Additional file 7 **Table S1 - Association between Nrf2 levels and clinicopathologic parameters in pancreatic tumors**. Data were available for all 57 patients, with the exception of perineural invasion (n = 55), vascular invasion (n = 54) and resection margin status (n = 52). Click here for file

Additional file 8 **Table S2 - Association between Keap1 levels and clinicopathologic parameters in pancreatic tumors**. Data were available for all 66 patients, with the exception of perineural invasion (n = 60), vascular invasion (n = 61) and resection margin status (n = 58). Click here for file

Additional file 9 **Table S3 - Oligonucleotide sequences**. A and B, Primers for gene sequencing. C, siRNA molecules. D, Primers for RTPCR analysis. Click here for file

Additional file 10 **Figure S7 - Isotype controls for Nrf2/Keap1 IHC staining in pancreatic tumors**. Click here for file
