# Supplementary figures and images for: Nrf2 is overexpressed in pancreatic cancer: implications for cell proliferation and therapy
Source: Mol Cancer. 2011 Apr 13;10:37. doi: 10.1186/1476-4598-10-37 (PMC3098205; doi:10.1186/1476-4598-10-37)

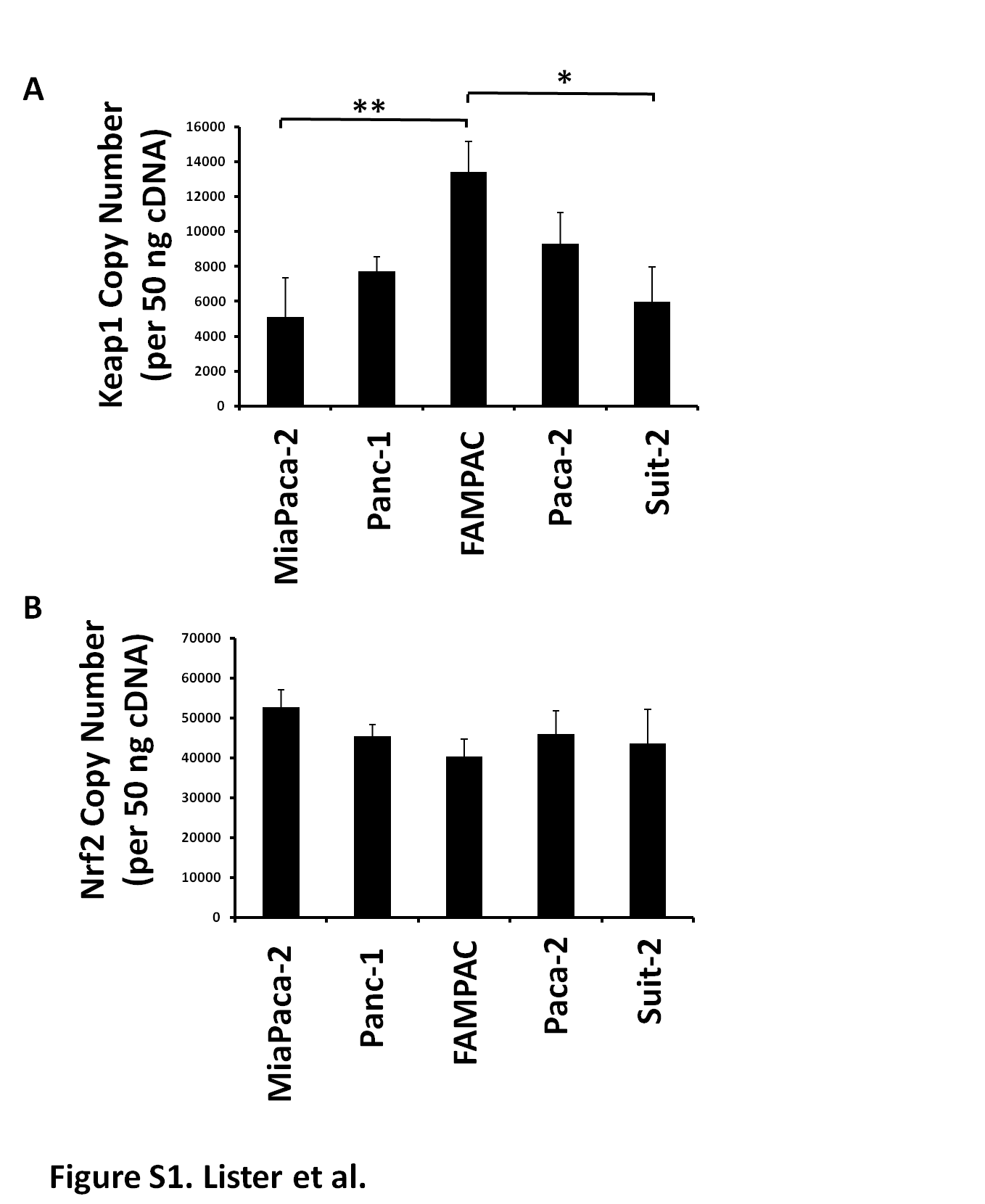

Supplement: Additional file 1 — Figure S1 - Quantification of Nrf2 and Keap1 mRNA abundance in pancreatic cancer cell lines. cDNA was synthesised from isolated RNA from five pancreatic cancer cell lines. Nrf2 and Keap1 mRNA levels were quantified by RTPCR using plasmids containing hNrf2 and hKeap1 cDNA as standards. [file 1476-4598-10-37-S1.PNG]

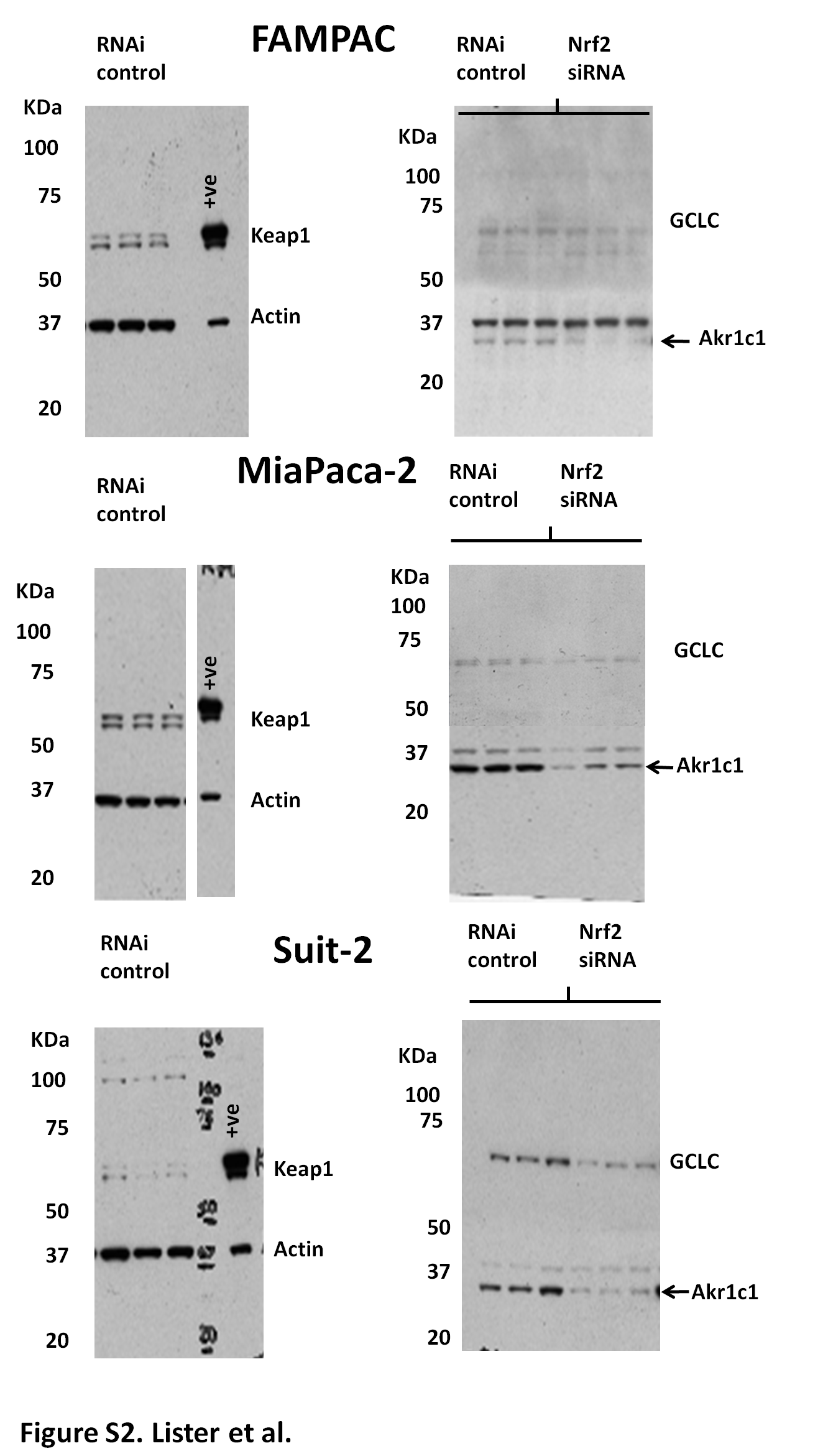

Supplement: Additional file 2 — Figure S2 - Immunoblots depicting the area between 20-100 KDa to demonstrate specificity of antibodies used in the study. Cells were transfected with 10 nM Nrf2-targeting siRNA, or Stealth RNAi control, for 96 h in three parallel experiments. A, Immunoblot detection of Keap1. Beta-actin is used as reference control. B, Immunoblot detection of GCLC and AKR1c1. [file 1476-4598-10-37-S2.PNG]

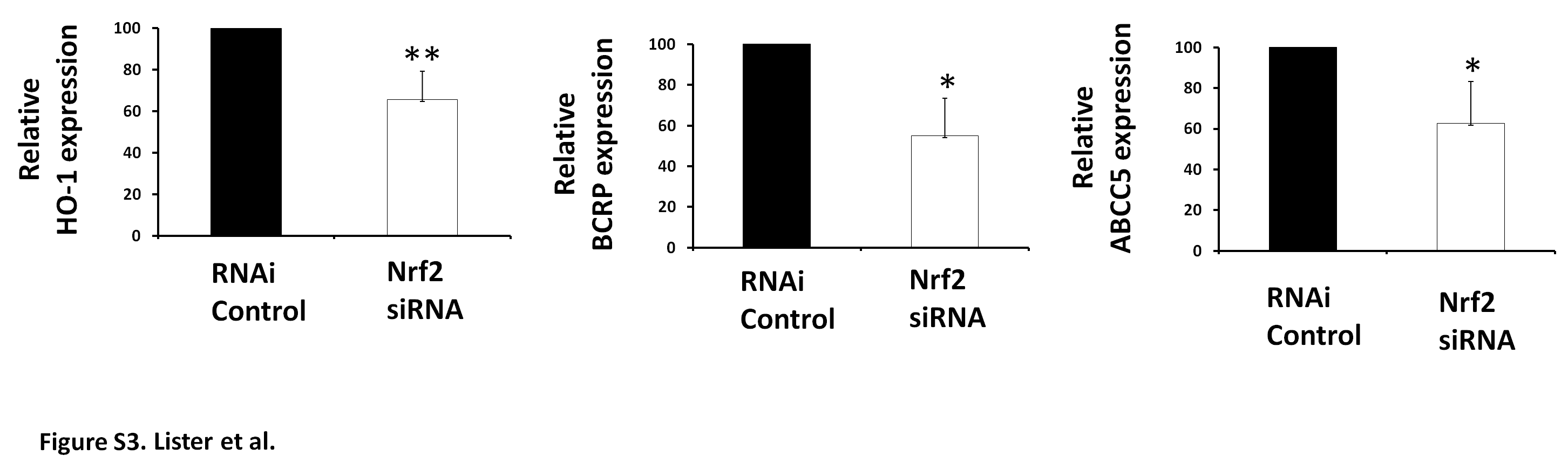

Supplement: Additional file 3 — Figure S3 - Quantification of HO-1, BCRP and ABCC5 mRNA levels in Suit-2 cells following siRNA depletion of Nrf2. Cells were transfected with 10 nM Nrf2-targeting siRNA, or Stealth RNAi control, for 96 h. mRNA levels were quantified by qRTPCR. GAPDH was used as a reference control. Data are the means ± S.D. of four discrete experiments. * = P < 0.05, ** = P < 0.01. [file 1476-4598-10-37-S3.PNG]

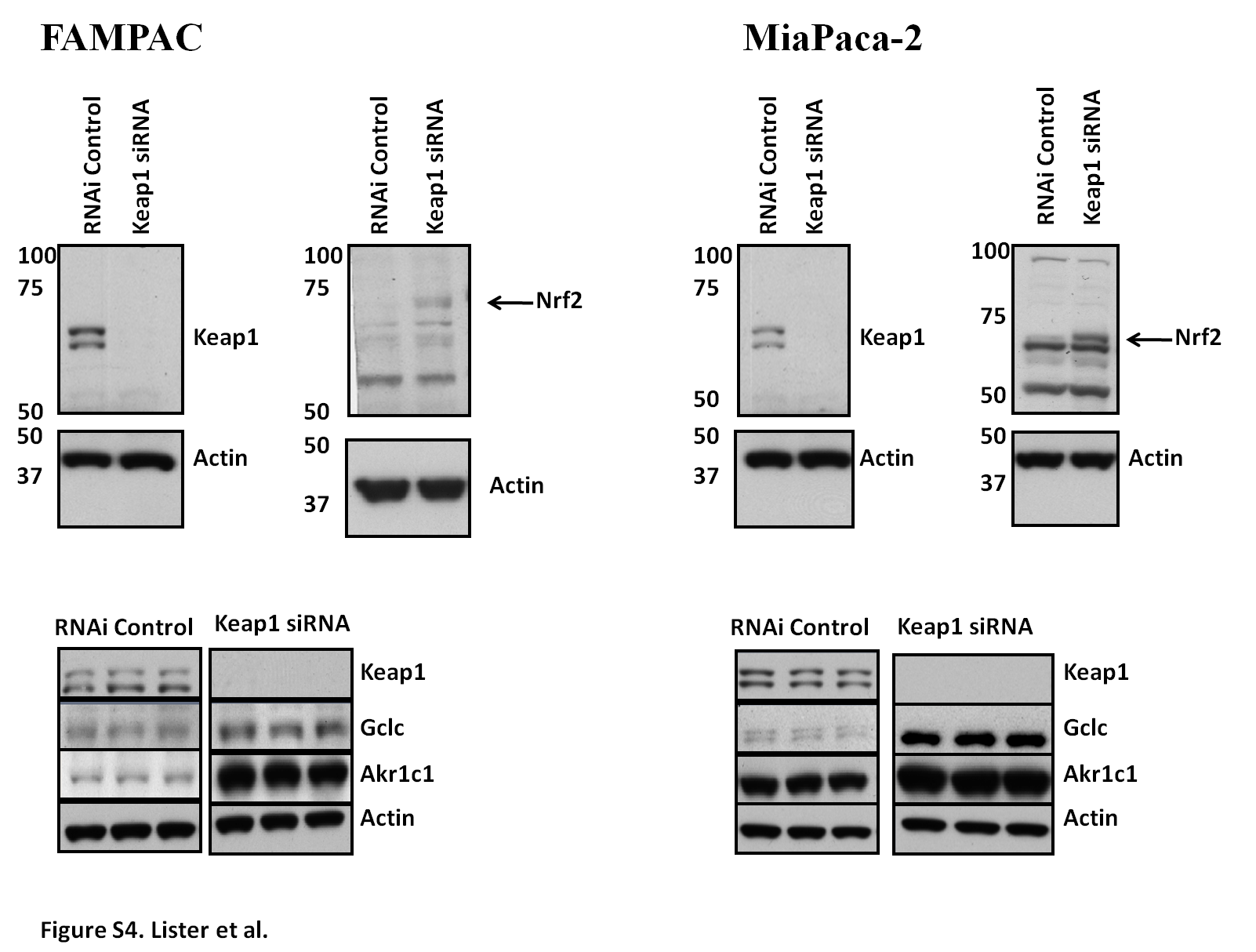

Supplement: Additional file 4 — Figure S4 - siRNA depletion of Keap1 in FAMPAC and Suit-2 cells. Cells were transfected with 10 nM Keap1-targeting siRNA, or Stealth RNAi control, for 96 h. Immunoblot detection of Keap1, Nrf2, GCLC and AKR1c1. Beta-actin is used as reference control. [file 1476-4598-10-37-S4.PNG]

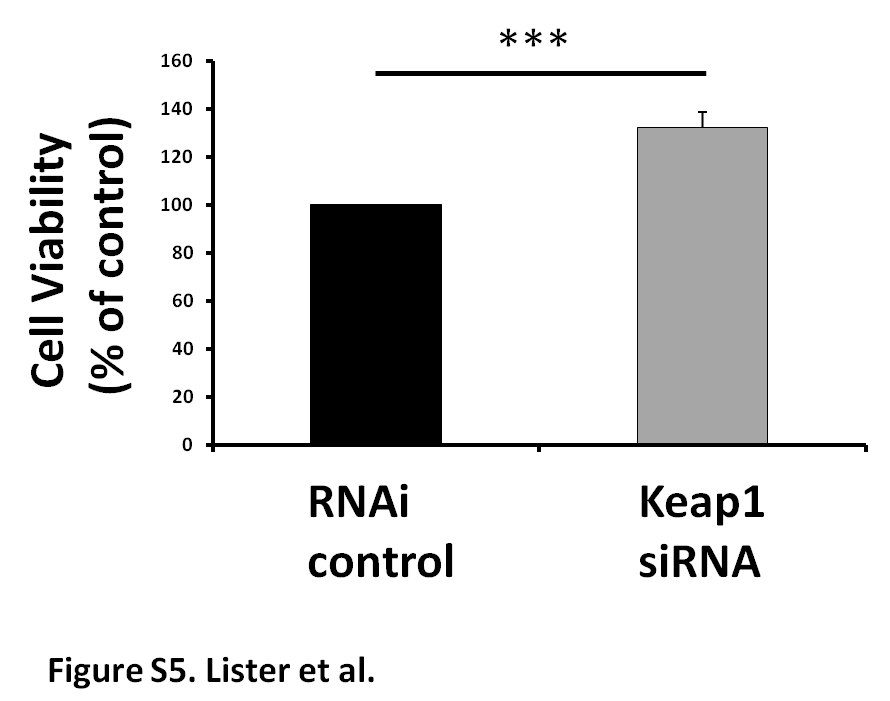

Supplement: Additional file 5 — Figure S5 - Effect of siRNA depletion of Keap1 on viability of FAMPAC cells. Cells were transfected with 10 nM Keap1-targeting siRNA, or Stealth RNAi control, for 120 h. Cell survival was measured using the MTS test. Data is shown as cell viability versus Stealth RNAi transfected control. *** = P < 0.001. [file 1476-4598-10-37-S5.PNG]

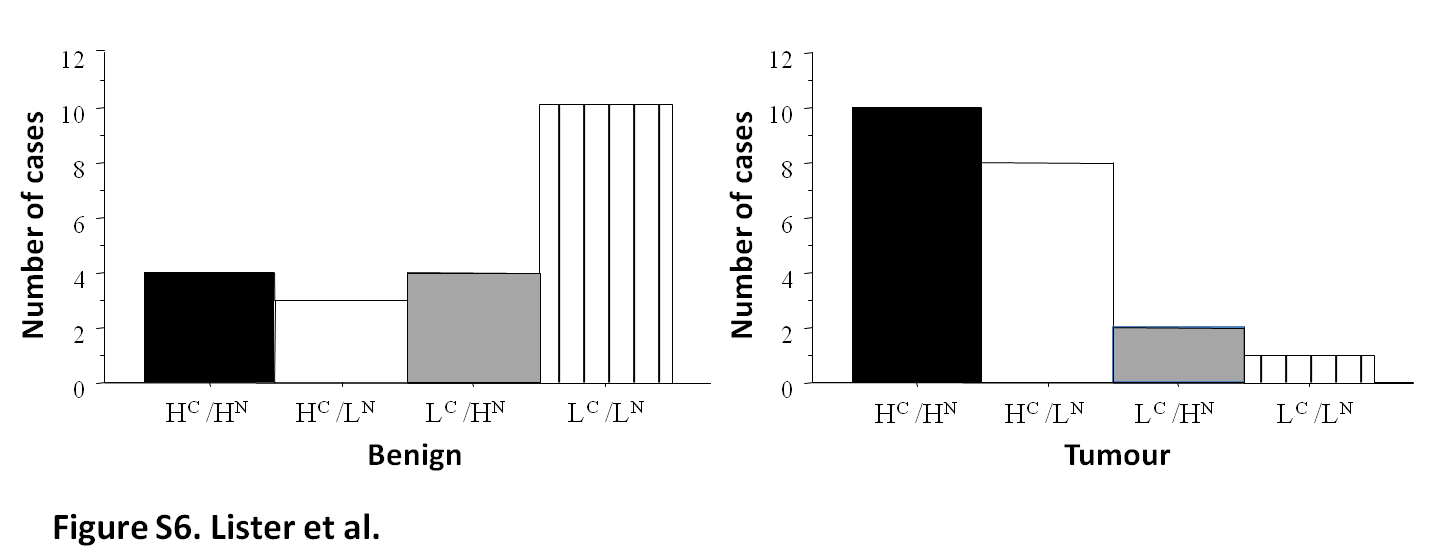

Supplement: Additional file 6 — Figure S6 - Distribution of Nrf2 staining in the cytoplasm and nucleus of tumors and matching benign cores. Histograms showing Nrf2 stained tissues (n = 21 tumor cases and matched benign tissue) categorised into 4 distinct groups, i.e. those containing: i) high cytoplasmic Nrf2 and high nuclear Nrf2 (HC/HN), ii) high cytoplasmic Nrf2 and low nuclear Nrf2 (HC/LN), iii) low cytoplasmic Nrf2 and high nuclear Nrf2 (LC/HN) and iv) low cytoplasmic Nrf2 and low nuclear Nrf2 (LC/LN). [file 1476-4598-10-37-S6.PNG]

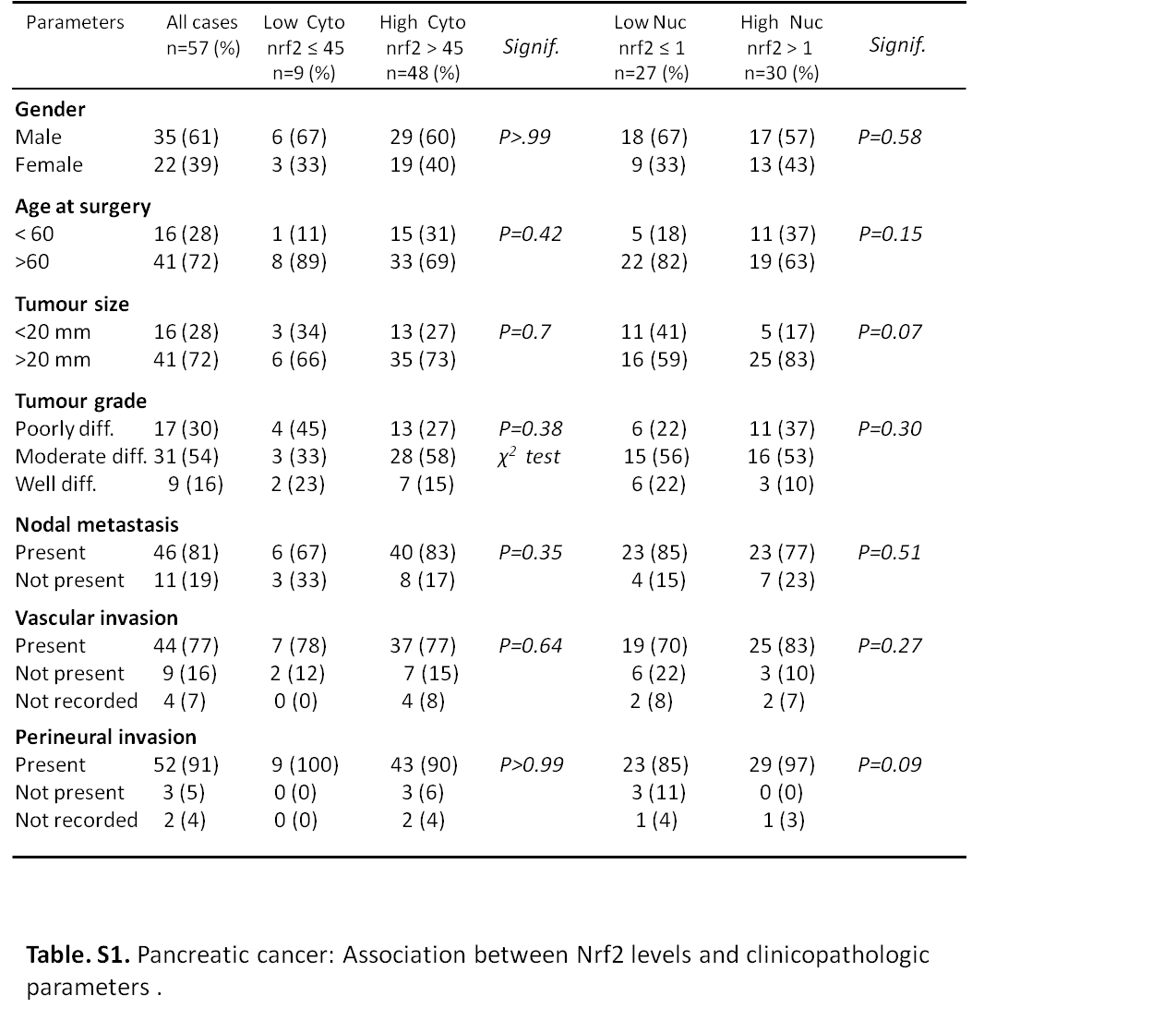

Supplement: Additional file 7 — Table S1 - Association between Nrf2 levels and clinicopathologic parameters in pancreatic tumors. Data were available for all 57 patients, with the exception of perineural invasion (n = 55), vascular invasion (n = 54) and resection margin status (n = 52). [file 1476-4598-10-37-S7.PNG]

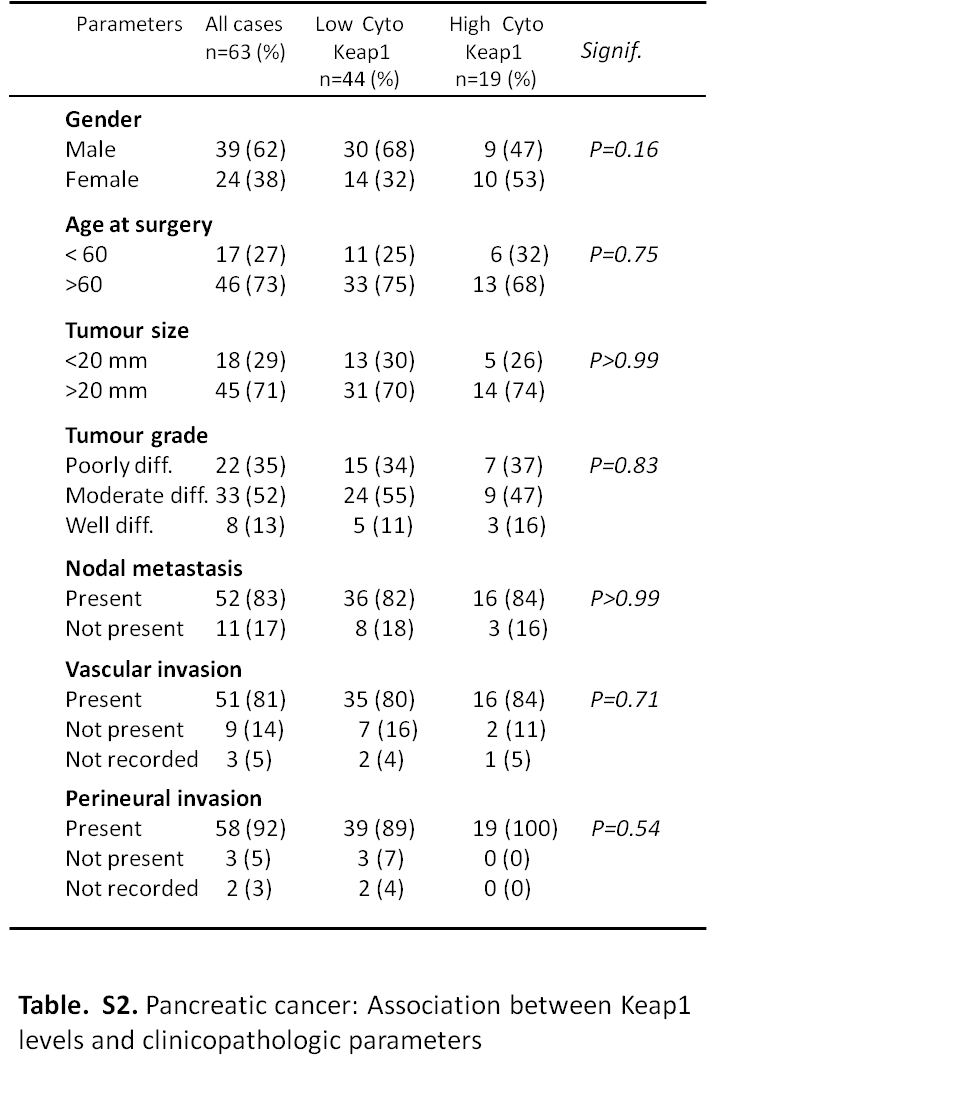

Supplement: Additional file 8 — Table S2 - Association between Keap1 levels and clinicopathologic parameters in pancreatic tumors. Data were available for all 66 patients, with the exception of perineural invasion (n = 60), vascular invasion (n = 61) and resection margin status (n = 58). [file 1476-4598-10-37-S8.PNG]

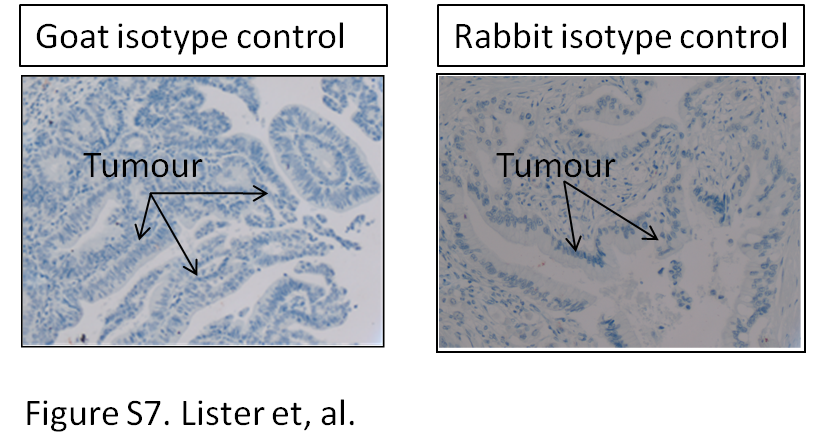

Supplement: Additional file 10 — Figure S7 - Isotype controls for Nrf2/Keap1 IHC staining in pancreatic tumors. [file 1476-4598-10-37-S10.PNG]
