# Supplementary material for: Nrf2 is overexpressed in pancreatic cancer: implications for cell proliferation and therapy
Source: Mol Cancer. 2011 Apr 13;10:37. doi: 10.1186/1476-4598-10-37 (PMC3098205; doi:10.1186/1476-4598-10-37)
Supplement: Additional file 9 — Table S3 - Oligonucleotide sequences. A and B, Primers for gene sequencing. C, siRNA molecules. D, Primers for RTPCR analysis. [file 1476-4598-10-37-S9.PDF]

Primer list for Keap1		
primer name	Forward primer (5' - 3')	Reverse primer (5' - 3')
exon 2	gcaaatggattctgcttcacctactt	tcaaactgtggagactacaccaccat
exon3	catcacaatgtacgcggttcctatat	ggcacagaatcaaaggtcactgacta
exon4	gatgaacctgtctctttaagggggaa	ggagagagagaagcttggactctatcagaa
exon4 (2)	gtccccatttttcttacgccc	gctcccctccctaccgtcccc
exon 5	gggagaggagagaggaaagg	agcaaaagcaaaagcagtcc
exon5 (2)	agtcaccttctctgcatg	ggctagtcaggactcttc
exon 6a	ctttctgtcccctgctcttg	gaagcctgctctttccacac
exon 6b	aaagcgggaatcatgtgc	ccagccctaagtcaaagagg
exon6 (2)	tgaccatcccttctgttcttc	ttcaggcctccagcccagagc
Primer list for Nrf2		
primer name	Forward primer (5' - 3')	Reverse primer (5' - 3')
exon2	cttcccaccatcaacagtg	cctgccataactttcccaag

Primers for RTPCR		
Gene	Forward primer (5' - 3')	Reverse primer (5' - 3')
Nrf2	gagagcccagtcttcattgc	ttggcttctggacttggaac
Keap1	cagattggctgtgtggagtt	gctgttcgcagtcgtacttg
GAPDH	cgaccactttgtcaagctca	aggggagattcagtgtggtg
HO-1	gccaggtgctcaaaaagatt	cctgcaactcctcaaaagagc
MRP5	tgacggaaatcgtgcggtcttggt	tgggaccaacaggcttcctggg
BCRP	ccattgcatcttggctgtcatggctt	tctctcaccctggggcttgtgg

siRNA molecules

Gene	manufacturer	code
Nrf2	dharmacon (Lafayette, USA)	D-003755-05
Keap1	invitrogen (Paisley, UK)	KEAP1-HSS114801(3RNA)
Negative universal control med	invitrogen (Paisley, UK)	46/2001
